# Portrait of DNA methylated genes predictive of poor prognosis in head and neck cancer and the implication for targeted therapy

**DOI:** 10.1038/s41598-021-89476-x

**Published:** 2021-05-11

**Authors:** Jessica Hier, Olivia Vachon, Allison Bernstein, Iman Ibrahim, Alex Mlynarek, Michael Hier, Moulay A. Alaoui-Jamali, Mariana Maschietto, Sabrina Daniela da Silva

**Affiliations:** 1 Department of Otolaryngology-Head and Neck Surgery, Lady Davis Institute for Medical Research and Segal Cancer Centre, Sir Mortimer B. Davis-Jewish General Hospital, McGill University, 3755 Côte Ste-Catherine Road, Montreal, QC H3T 1E2 Canada; 2 Segal Cancer Centre of the Lady Davis Institute for Medical Research, Sir Mortimer B. Davis-Jewish General Hospital, McGill University, Montreal, QC Canada; 3 Department of Structural and Functional Biology, Institute of Biology, Universidade Estadual de Campinas (UNICAMP) and Boldrini Children’s Center, Campinas, Sao Paulo Brazil

**Keywords:** Cancer, Predictive markers

## Abstract

In addition to chronic infection with human papilloma virus (HPV) and exposure to environmental carcinogens, genetic and epigenetic factors act as major risk factors for head and neck cancer (HNC) development and progression. Here, we conducted a systematic review in order to assess whether DNA hypermethylated genes are predictive of high risk of developing HNC and/or impact on survival and outcomes in non-HPV/non-tobacco/non-alcohol associated HNC. We identified 85 studies covering 32,187 subjects where the relationship between DNA methylation, risk factors and survival outcomes were addressed. Changes in DNA hypermethylation were identified for 120 genes. Interactome analysis revealed enrichment in complex regulatory pathways that coordinate cell cycle progression (*CCNA1, SFN, ATM, GADD45A, CDK2NA, TP53, RB1* and *RASSF1*). However, not all these genes showed significant statistical association with alcohol consumption, tobacco and/or HPV infection in the multivariate analysis. Genes with the most robust HNC risk association included *TIMP3, DCC, DAPK, CDH1, CCNA1, MGMT, P16, MINT31, CD44, RARβ*. From these candidates, we further validated CD44 at translational level in an independent cohort of 100 patients with tongue cancer followed-up beyond 10 years. CD44 expression was associated with high-risk of tumor recurrence and metastasis (*P* = 0.01) in HPV-cases. In summary, genes regulated by methylation play a modulatory function in HNC susceptibility and it represent a critical therapeutic target to manage patients with advanced disease.

## Introduction

Head and neck cancer (HNC), the 6th common cancer worldwide, is characterized by high incidence of local tumor invasion and metastatic spread^1,2^. Despite of the advances in diagnosis and treatment modalities, high mortality rates rank HNC among the most aggressive cancers. This aggressiveness is contributed by the high loco-regional relapse seen at early stages, which is worsened by the heterogeneous nature of the disease involving a variety of histological tumor subtypes and affecting diverse anatomical sites^3^. Historically, the traditional risk factors for HNC include excessive tobacco smoking, alcohol consumption, and infection by human papillomavirus (HPV). Additional factors have been identified to enhance individual susceptibility to HNC, in particular, genetic abnormalities impacting on cell proliferation, differentiation features, cell cycle checkpoints, angiogenesis and tumor metabolism^4–8^. Furthermore, deregulation of epigenetic machinery such as DNA methylation, nucleosome positioning, histone modifications and non-coding RNAs have been reported to contribute to enhanced individual susceptibility to HNC with direct influence on gene activities^9^.

DNA methylation is the major epigenetic alteration characterized by addition or removal of a methyl group (CH3) referred as hypermethylation of the CpG islands or global hypomethylation, respectively^10^. DNA hypomethylation has been associated with chromosomal instability as well as activation of proto-oncogenes, while DNA hypermethylation has been involved in repressing tumor suppressor genes and genomic instability often impacting on tumor initiation and progression^9,10^. The reversible nature of epigenetic aberrations has led to the promising benefit of epigenetic therapy for cancer prevention and management^11^. However, DNA methylation status vary according HNC subtypes, differentiation features, anatomic involvement^12,13^, HPV status^14^, smoking habits^9^ and geographic distribution^15^. Therefore, identifying crucial genes that are susceptible to DNA hypermethylation-induced gene silencing is becoming critical to tailor the utility of methylation modifiers to individual cancer types.

Here, we systematically reviewed published papers addressing epigenetic alterations, particularly DNA hypermethylation, in relation to individual susceptibility to HNC, as well as HNC progression and prognosis. We confirmed using a multivariate analysis the clinical relevance of 10 most common alterations as independent risk factors for HNC progression. Furthermore, we used a network-based analysis to prioritize putative molecular interactions and validate the candidates by protein expression in a cohort of HNC with long-term follow-up. Last, we discussed the potential of relevant FDA-approved drugs as alternative therapeutics for invasive HNC.

## Materials and methods

### Data search

**Figure 1:**
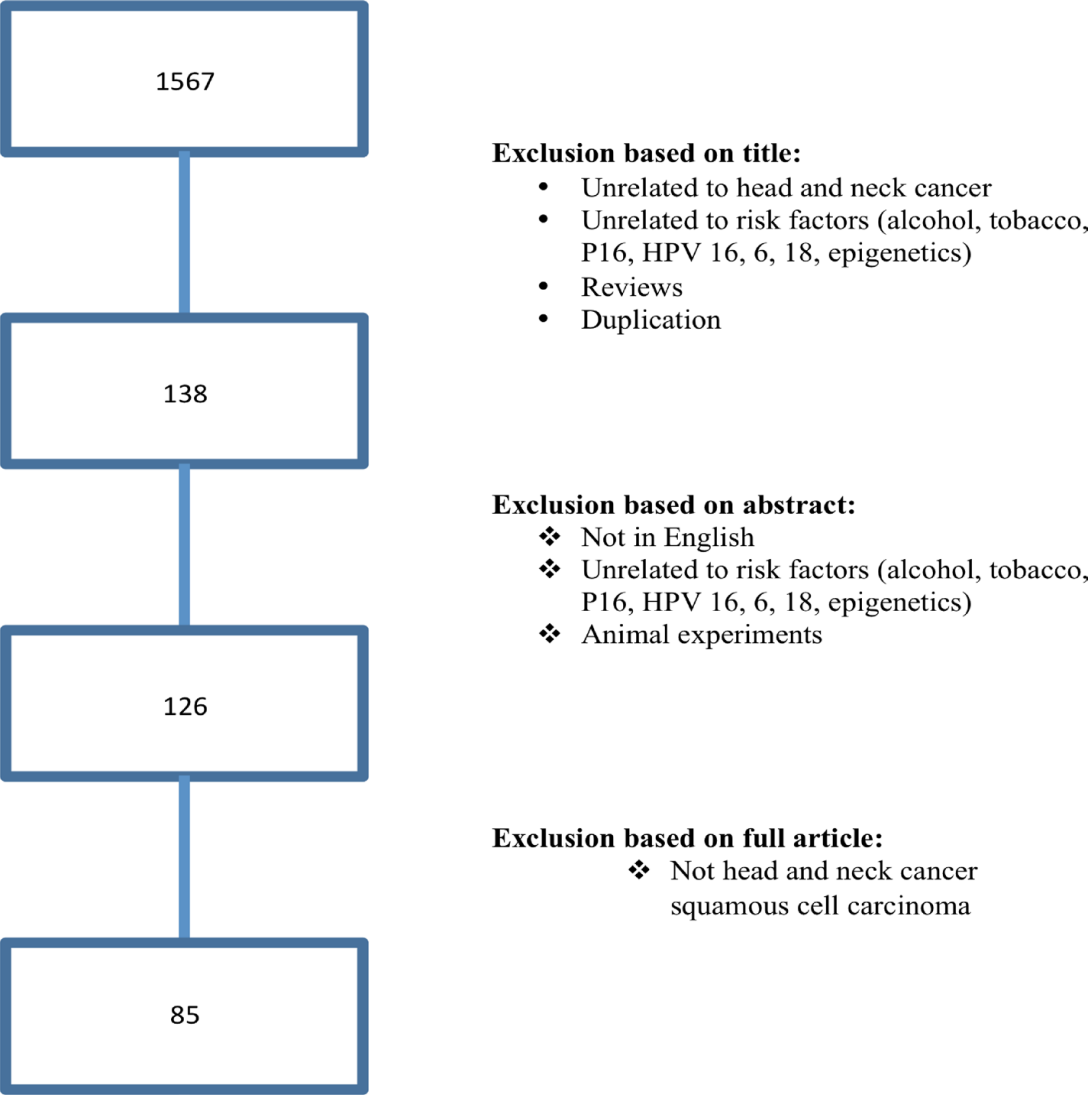
Flow diagram of search and study selection process. Following the guidelines of the Meta-analysis of Observational Studies in Epidemiology group (MOOSE), we performed a broad and sensitive search on online databases to identify the studies that examined associations between DNA methylation and HNC associated with risk factors (alcohol, tobacco and HPV infection). A systematic literature search for relevant studies up to June 2020. In this study, we considered the clinical endpoints overall survival (OS) and disease specific survival (DFS) as acceptable outcomes. The prognostic value was demonstrated using hazard ratio (HR) with 95% confidence interval (CI).

The study followed the protocol recommended by Cochrane Handbook for Systematic Reviews of Interventions (https://training.cochrane.org). In brief, we conducted this systematic literature review using online platforms: PubMed, Wiley Online Library, EMBASE, Web of Science, Scopus, and Cochrane databases between January 2008 and June 2020. The tested hypothesis was to establish the associations between epigenetic alteration and HNC risk. The search strategy focused on key words including their abbreviation, truncations, synonyms, and subsets for search, such as: “head and neck neoplasms” or “facial neoplasms” or “head and neck cancer” or “oral cancer” or “tongue cancer” or “mouth cancer” or the codes described in the International Classification of Diseases for Oncology (ICD-O) for Head and Neck Tumors (https://www.who.int); and “epigenetics” or “epigenomics” or “methylation” or “histone modification” or “non-coding RNA” or “ncRNA” and “risk factors” or “smoke” or "tobacco" or “alcohol” or “HPV”. Searches in Gene Expression Omnibus (GEO, www.ncbi.nlm.nih.gov/geo/) and ArrayExpress (www.ebi.ac.uk/arrayexpress) repositories were also performed. We designed this strategy for a sensitive and broad search (Fig. 1). Additional relevant studies from the reference lists were also included in the analysis. Two librarian experts in systematic review methods hand searched the references list to find additional articles.

### Inclusion and exclusion criteria

This study did not include non-English manuscripts, single case reports, editorial letters, and reviews of literature. It was also excluded cross-sectional studies that addressed associations with alcohol, tobacco and HPV status without specifically examining associations with epigenetic alteration. Studies using only preclinical models were also excluded. Then, the following inclusion criteria were required to be eligible in this systematic review: (1) human case–control studies; (2) clinical studies related to the DNA methylation and HNC risk factors; (3) methylation sequencing and array methods were excluded; 4) when the same research group was identified, publications were further investigated to eliminate duplications or samples overlap. The outcomes were further explored considering Hazard ratio (HR) with confidence of interval (CI) and *P* value < 0.05. Papers that fulfilled these criteria were processed for data extraction and the discrepancies were solved by discussion.

### Data extraction and quality assessment

A standardized form adapted from Dutch Cochrane Centre (https://netherlands.cochrane.org) for epidemiological studies was used to extracted the date and its included: (a) clear definition of risk factors (alcohol, tobacco and HPV status); (b) clear definition of the molecular assay used for the measurement of epigenetic alteration (e.g. quantitative real time polymerase chain reaction (qRT-PCR), methylation-specific PCR (MSP); (c) clear definition of cut-off, (d) definition of the anatomical site; e) definition of the target population (country where the study took place). To be qualified, all the criteria had to be mentioned in the manuscript; otherwise, the study was recorded and excluded from the systematic review.

In detail, data extracted from the final eligible articles include: first author, year of publication, impact factor of the journal publication, the country of origin, study design, population studied, subjects’ ethnicity, the number of cases, cancer types, source of control, epigenetic profiling, specimen, anatomic location, risk, HR and follow-up. The methodological quality and risk of bias was assessed by the Quality Assessment of Diagnostic Accuracy Studies 2 (QUADAS-2) score system.

### Network and enrichment analyses

The list of epigenetic alterations, focusing on DNA hypermethylation, was submitted to GSEA to search for enriched biological processes (Gene Onology) and cellular pathways (KEGG) using FDR < 0.05 or top 50 as parameters^16,17^. The SIGnaling Network Open Resource 2.0 (SIGNOR 2.0), a public repository that stores almost 23,000 manually annotated causal relationships between proteins and other biologically relevant entities (chemicals, phenotypes, complexes and others) was used to construct a protein–protein interaction (PPI) network using all types of interactions and score 0.1 as parameters^18^.

### Validation—study population

A retrospective study was performed by analyzing data from 100 patients with primary HNC diagnosed and treated at the Department of Otolaryngology—Head and Neck Cancer at the Jewish General Hospital (McGill University) (Supplementary Table 1). The eligibility criteria included previously untreated patients with diagnosis of HNC submitted to the treatment in a single institution. This study was carried out with the approval of the Human Research Ethics Committee of the Jewish General Hospital (JGH)—McGill University, Canada (protocol#11–093) and informed consent was obtained from all subjects. Strengthening the reporting of observational studies (STROBE Statement) was used to ensure appropriate methodological guidelines and regulations.

### Immunohistochemistry (IHC) analysis

IHC reaction and analysis were carried out as we previously described^19^. In brief, the incubations with the primary antibody anti-CD44 (Dako, 1:100) diluted in PBS were made overnight at 4 °C. Positive and negative controls were included in all reactions. IHC reactions were performed in duplicates to represent different levels tissues levels in the same lesion. The second slide was 25–30 sections deeper than the first slide, resulting in a minimum of 300 µm distance between sections representing fourfold redundancy with different cell populations for each tissue. IHC scoring was blinded to the outcome and clinical aspects of the patients. Cores were scanned in 10× power field to settle on the foremost to marked area predominant in a minimum of 10% of the neoplasia. IHC reaction was considered as positive if of a clearly visible dark brown precipitation occurred. IHC analysis was semi-quantitative considering the percentage and intensity of staining as: 0 (no detectable reaction or little staining in < 10% of cells), 1 (weak but positive IHC expression in > 10% of cells) and 2 (strong positivity in > 10% of cells). The percentage of CD44 positive was calculated with an image computer analyzer (Kontron 400, Carl Zeiss, Germany)^19^.

### Data analysis

The statistical analyses were performed using the STATA 12.0 statistical software (STATA Corporation, College Station, TX, USA) as we previously described^19^. The pooled parameters sensitivity, specificity, diagnostic hazard ratio (HR), and their 95% CIs were calculated to evaluate the overall diagnostic accuracy and the correlation between IHC status and HNC comparing high and low-risk patients. Statistical analysis considered the weighted effect, and the effect size was adjusted.

## Results

### Overview of the included studies

Following the search protocol and screening strategy, it was identified 1567 manuscripts. After exclusion of duplicates studies and manuscripts unrelated to epigenetic alteration or cancer, and reviews, 138 articles were retrieved for the title and abstract. Additional 12 studies were excluded, since they were either only abstracts or irrelevant to risk factors in HNC, leaving 126 studies for further full-text analysis (Fig. 1)^19–103^. Titles and abstracts retrieved through this search were screened by three of the authors (JH, OV, AB) and after a careful reading of the texts, 41 studies were removed due to the lack of information regarding survival analysis. Finally, we had 85 studies involving 32,187 subjects where the relationship between DNA hypermethylation and risk factors for HNC progression were analyzed (Table 1). QUADAS-2 evaluation analysis showed that all studies had relative elevated scores, indicating a comparatively high quality of the researchers included in this study. The median impact factor of these publications was 3.798 (range 0.652 to 9.238).

**Table 1:** Hypermethylation in genes associated with risk factors in patients with head and neck squamous cell carcinoma in the 85 identified studies.

Author	Impact factor	Type of Study	Population	Sample size	Anatomic location	Epigenetic alteration	Assay
Cordeiro-Silva et al.	1.698	Case–Control	Brazil	70/41	OC	CDKN2A, SFN, EDNRB, RUNX3	MSP
Sanchez-Cespedes et al.	9.329	Retrospective	USA	95	HNC	CDKN2A, MGMT, GSTP1, DAPK	MSP
Markowski et al.	1.554	Retrospective	Poland	21	larynx	HIC1	qRT-PCR
Virani et al.	3.362	Retrospective	USA	346	HNC	CCNA1, NDN, CD1A, DCC, CDKN2A, GADD45A	MSP
Shintani et al.	1.521	Retrospective	Japan	17	OC	CDKN2A	MSP
Agnese et al.	9.269	Retrospective	Italy	173	HNC	CDKN2A	MSP
Kawakami et al.	2.915	Retrospective	Japan	104	OP	CDKN2A	MSP
Ruesga et al.	5.992	Prospective	Spain	175	OC	CDKN2A	MSP
Zheng et al.	4.125	Case–Control	USA	208/ 245	HNC	CDKN2A	qRT-PCR
Sun et al.	3.234	Prospective	USA	197	OC and OP	CDKN2A, CCNA1, DCC, TIMP3, MGMT, DAPK, MINT31	MSP
Calmon et al.	2.805	Prospective	Brazil	43	HNC	CDKN2A, DAPK1, CDH1, ADAM23	MSP
Langevin et al.	5.108	Case–Control	USA	92/ 92	HNC	FGDA, SERPINF1, WDR39, IL27, HYAL2, PLEKHA6	qRT-PCR
Zhang et al.	3.234	Retrospective	Japan	10	OP	LCR	MSP
Hasegawa et al.	5.979	Retrospective	Israel	80	HNC	CDKN2A, DAPK, CDH1, RASSF1A	MSP
Misawa et al.	3.081	Retrospective	Japan	100	HNC	CDKN2A	MSP
Marsit et al.	5.649	Retrospective	USA	340	HNC	CDH1	MSP
Dikshit et al.	5.649	Retrospective	Italy	235	HNC	MGMT, DAPK, CDKN2A, CDH1	MSP
Farias et al.	3.025	Retrospective	Brazil	75	HNC	CDKN2A	MSP
Wong et al.	5.417	Prospective	China	73	HNC	P15, CDKN2A	MSP
Smith et al.	5.531	Retrospective	USA	137	HNC	CCNA1, MGMT, DCC, CDKN2A	MSP
Shaw et al.	3.93	Retrospective	UK	48	OC	CDKN2A, CYGB, CDH1, TMEFF2	MSP
Wong et al.	0.795	Retrospective	Taiwan	64	OC	DAPK, MGMT	MSP
Dong et al.	1.859	Prospective	China	30	OC	CDKN2A	MSP
Prez-Sayans et al.	1.553	Retrospective	Spain	68	OC	CDKN2A	MSP
Tran et al.	1.859	Prospective	Vietnam	36	OC	CDKN2A, RASSF1A	MSP
Kaur et al.	5.531	Prospective	India	92	OC	DCC, EDNRB, CDKN2A, KIF1A	MSP
Virani et al.	3.135	Retrospective	USA	98	HNC	CCNA1, NDN	MSP
Nakagawa et al.	3.523	Prospective	Japan	58	OC	LRP1B	qRT-PCR
Morandi et al.	1.252	Retrospective	Italy	48	OC	GP1BB, ZAP70, KIF1A, CDKN2A, CDH1, miR137, miR375	MSP
Taioli et al.	3.362	Retrospective	USA	88	OC and OP	MGMT, CDKN2A, RASSF1	MSP
Parfenov et al.	9.423	Prospective	USA	129	HNC	BARX2, IRX4, SIM2	qRT-PCR
Lee et al.	7.429	Retrospective	Taiwan	40	OC	BEX1, LDOC1	MSP
Chang et al.	5.649	Prospective	China	90	HNC	P15	MSP
Schussel et al.	1.186	Prospective	Brazil	47	OC	DACT1, DACT2	MSP
Wilson et al.	5.108	Prospective	USA	6	HNC	CDH1	MSP
Nayak et al.	2.272	Retrospective	USA	124	HNC	TIMP3, DAPK	MSP
Ogi et al.	8.738	Retrospective	Japan	96	OC	CDKN2A, P15, P14, DCC, DAPK, MINT1, MINT2, MINT27, MINT31	qRT-PCR
Colacino et al.	3.234	Retrospective	USA	68	HNC	GRB7, CDH11, RUNX1T1, SYBL1, TUSC3, SPDEF, RASSF1, STAT5A, MGMT, ESR2, JAK3, HSD17B12	MSP
Langevin et al.	4.327	Retrospective	USA	154	HNC	DKK1, ZCCHC14, MARCH4, ANKRD33B, SLC6A5, INPP5A, ATAD3C, PWWP2B, SAFB2, GABRA1, KCNQ1, PTHLH, ARHGEF2, CIT, SH3BP5	qRT-PCR
Misawa et al.	8.738	Prospective	Japan	100	HNC	GALR1	MSP
Langevin et al.	3.607	Retrospective	USA	82	OC	GABBR1	qRT-PCR
Bebek et al.	5.985	Prospective	USA	42	HNC	MDR1, IL8, RARB, TGFBR2	MSP
Ohta et al.	1.262	Prospective	Japan	44	OC	CDKN2A, P14ARF	MSP
Furniss et al.	4.125	Retrospective	USA	303	HNC	LRE1	MSP
Zhao et al.	2.301	Retrospective	China	41	nasopharynx	GALC	qRT-PCR
Hsiung et al.	4.125	Case–Control	USA	278/ 526	OC and OP	MTHFR	MSP
Sinha et al.	3.135	Prospective	India	38	OC	CDKN2A	MSP
Khor et al.	2.244	Prospective	Malaysia	20	OC	CDKN2A, DDAH2, DUSP1	MSP
O'Regan et al.	2.769	Prospective	Ireland	24	OC and OP	CDKN2A	MSP
Weiss et al.	4.722	Retrospective	Germany	86	HNC	TIMP3, CDH1, CDKN2A, DAPK1,TCF21, CD44, MLH1, MGMT, RASSF1, CCNA1, LARS2, CEBPA	MSP
Sun et al.	8.738	Retrospective	USA	197	HNC	CCNA1, MGMT, MINT31	MSP
Supic et al.	4.602	Prospective	Serbia	96	OC	CDKN2A, RASSF1A, DAPK, CDH1, MGMT, hMLH1, WIF1, RUNX3	MSP
Weiss et al.	3.562	Prospective	Germany	74/ 41	HNC	TCF21	MSP
Ai et al.	5.485	Retrospective	USA	100	HNC	CDKN2A	MSP
El-Naggar et al.	6.501	Retrospective	USA	46	HNC	CDKN2A	MSP
González-Ramírez et al.	3.607	Case–Control	Mexico	50/200	OC	MLH1	MSP
Gemenetzidis et al.	3.234	Prospective	UK	75	HNC	FOXM1	qRT-PCR
Ishida et al.	3.607	Prospective	Japan	49	OC	CDKN2A, P14, RB1, P21, P27, PTEN, P73, MGMT, GSTP	MSP
Righini et al.	8.738	Prospective	France	90	HNC	TIMP3, CDH1, CDKN2A,MGMT, DAPK, RASSF1	MSP
Subbalekha et al.	3.607	Case–Control	Thailand	69/37	OC	LINE1	MSP
Dong et al.	8.738	Prospective	USA	46	OP	RASSF1A	MSP
Ovchinnikov et al.	2.884	Case–Control	Australia	143/31	HNC	RASSF1A, DAPK1, CDKN2A	MSP
Demokan et al.	2.760	Prospective	Turkey	77	HNC	CDKN2A	MSP
Kresty et al.	9.329	Retrospective	USA	26	OC	CDKN2A, P14	MSP
Marsit et al.	5.334	Prospective	USA	68	HNC	HGF, FGF, ATP10A, NTRK3, ZAP70, GP1BB, SRC, EGF, EPHA2	MSP
Mielcarek-Kuchta et al.	2.926	Prospective	Poland	53	OC and OP	CDKN2A, CDH1, ATM, FHIT, RAR	MSP
Steinmann et al.	2.301	Prospective	Germany	54	HNC	RASSF1A, CDKN2A, MGMT, DAPK, RARß, MLH1, CDH1, GSTP1, RASSF2, RASSF4, RASSF5, MST1, MST2, LATS1, LATS2	MSP
Tan et al.	5.569	Prospective	France	42	HNC	CDKN2A, CCNA1, DCC	MSP
Pannone et al.	1.718	Prospective	Italy	64	OC and OP	CDKN2A	MSP
Kulkarni et al.	3.607	Prospective	India	60	OC	CDKN2A, DAPK, MGMT	MSP
Huang et al.	2.207	Case–Control	Taiwan	31/40	OC	SOX1, PAX1, ZNF582	MSP
Misawa et al.	1.736	Prospective	Japan	46	HNC	COL1A2	MSP
Koscielny et al.	0.492	Prospective	Germany	67	HNC	CDKN2A	MSP
Miracca et al.	5.569	Prospective	Brazil	47	HNC	CDKN2A	MSP
Rosas et al.	9.329	Retrospective	USA	30	HNC	CDKN2A, DAPK, MGMT	MSP
Roh et al.	8.738	Prospective	USA	353	HNC	CDKN2A, DCC, EDNRB, KIF1A	MSP
Supic et al.	2.495	Retrospective	Serbia	76	OC	RUNX3, W1F1	MSP
Sharma et al.	2.495	Prospective	India	73	HNC	CYP1A1, CYP2A13, GSTM1	MSP
Choudhury et al.	3.234	Retrospective	India	116	HNC	CDKN2A, DAPK, RASSF1, BRAC1, GSTP1, CDH1, MLH1, MINT1, MINT2, MINT31	MSP
Park et al.	4.444	Prospective	USA	22	OP	LCR	MSP
Balderas-Loaeza et al.	5.531	Prospective	Mexico	62	OC	LCR	MSP
Marsit et al.	5.531	Retrospective	USA	350	HNC	SFRP1, SFRP2, SFRP4, SFRP5	MSP
Ayadi et al.	1.826	Retrospective	Tunisia	44	nasopharynx	CDKN2A, DLEC1, BLU, CDH1	MSP
Puri et al.	0.933	Retrospective	USA	51	HNC	MLH1, MGMT, CDKN2A	MSP
Gubanova et al.	8.738	Prospective	USA	40	OP	SMG1	qRT-PCR

MSP: methylation specific PCR; OC: oral cancer; OP: oropharyngeal cancer; HNC: head and neck cancer.

Of the 85 articles exploring DNA methylation and risk factors (including tobacco use, alcohol abuse, and HPV positivity) in HNC, 30 (35.3%) studies focused on North Americans populations followed by Japanese (n = 10; 11.8%), Brazilian (n = 5; 5.9%) and India population (n = 5; 5.9%). DNA methylation was widely analyzed by MSP of specific genes in 74 (87.1%) studies. The remaining researches used qRT-PCR as method (11 studies; 12.9%). The anatomic location in head and neck cancer was predominantly mixed (44 studies; 51.8%) followed by oral cavity (n = 27; 31.8%) and oral cavity mixed with oropharyngeal cases (n = 6; 7.1%). A total of 37 (46.5%) of the 85 articles only measured DNA methylation of a single gene (Table 1).

### DNA methylation associated with cancer risk in HNC

**Figure 2:**
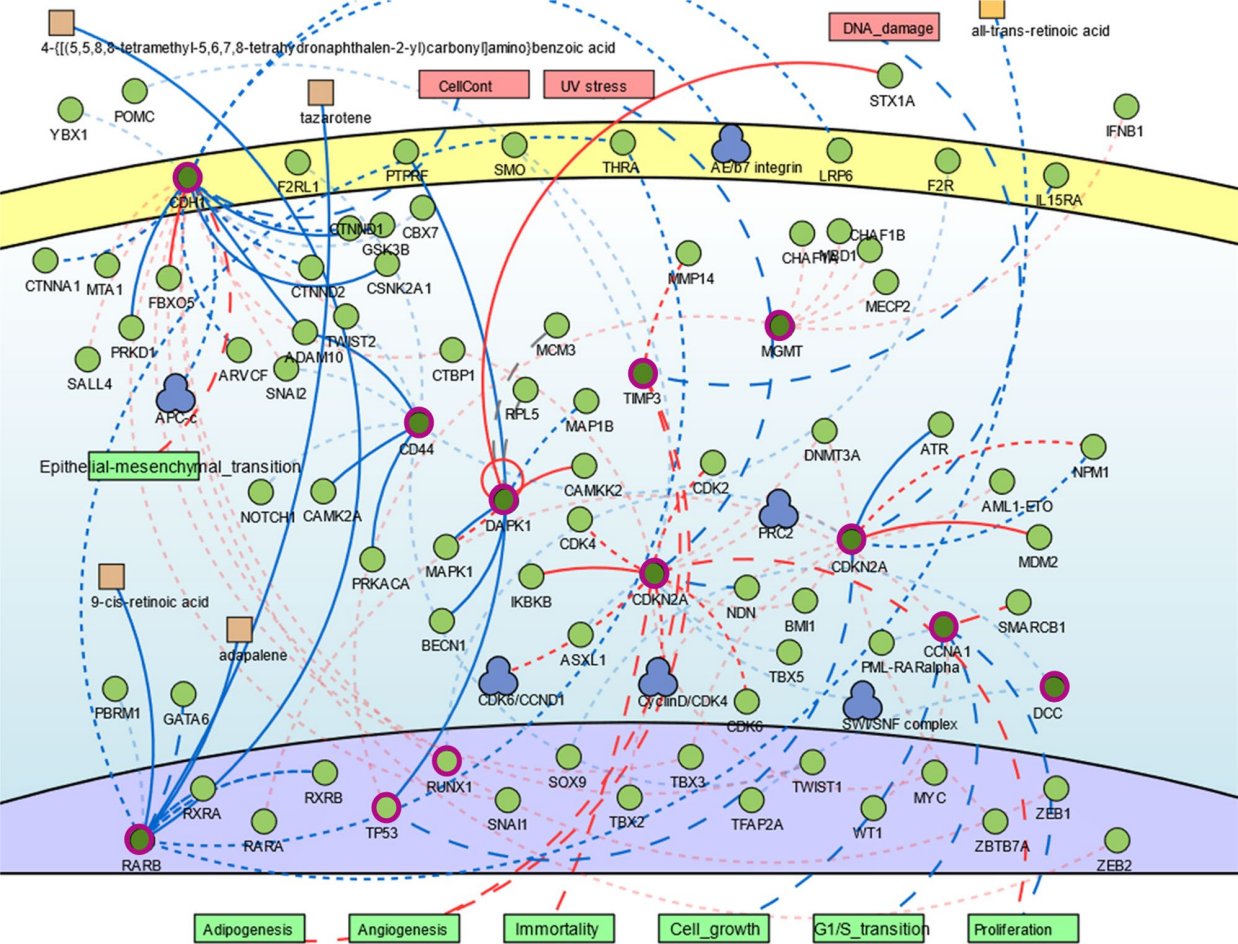
Genomic network analysis showing the central role of genes related with cell cycle pathway. Genes hypermethylated (circled in pink) from different studies were involved into common biological processes suggesting that they work together. PPI analysis pointed to external stimulus, such as DNA damage, UV stress, all-trans-retinoic acid that could activate a cellular signalization to epithelial-mesenchymal transition (EMT), adipogenesis, angiogenesis, immortality, cell growth, cell cycle and proliferation. Image done using the public repository SIGnaling Network Open Resource 2.0 (SIGNOR 2.0).

Changes in DNA hypermethylation were identified for 120 genes (Table 1). These genes are enriched for biological processes related to cell proliferation and death, response to stimulus (including drugs), metabolism, and cellular motility and differentiation (Supplementary Table 2). Even though these genes came from different studies, the interactome analysis showed that some of these genes, such as *CCNA1, SFN, ATM, GADD45A, CDKN2A, TP53, RB1* and *RASSF1* are involved into common biological processes suggesting that they work together (Fig. 2). Thus, we verified the cellular pathways where the regulatory genes play critical role in the signaling networks, including p53, Wnt, MAPK and ErbB tyrosine kinase receptor signaling, as well as cytochrome P450-associated xenobiotic metabolism (Supplementary Table 3).

In the multivariate analysis, not all the 120 genes showed a significant correlation with alcohol, tobacco and/or HPV status. Rather, only the hypermethylation of *TIMP3, DCC, DAPK1, CDH1, CCNA1, MGMT, P16 (CDKN2A), MINT, CD44, RARβ* were associated with these known risk factors in progressive HNC. According to GSEA (Supplementary Table 2), five of these genes belong to four families sharing similar homology or biochemical activity: tumor suppressors (*CDH1* and *CDKN2A*), protein kinase (*DAPK1*), cell differentiation markers (*CDH1* and *CD44*) and transcriptional factor (*RARβ*). These ten genes were submitted to signaling network analysis revealing a protein-to-protein interaction (PPI) that pointed to external stimulus, such as DNA damage, UV stress, all-trans-retinoic acid that could activate a cellular signalization to epithelial-mesenchymal transition, adipogenesis, angiogenesis, immortality, cell growth, cell cycle (G1S transition) and proliferation (Fig. 2).

**Figure 3:**
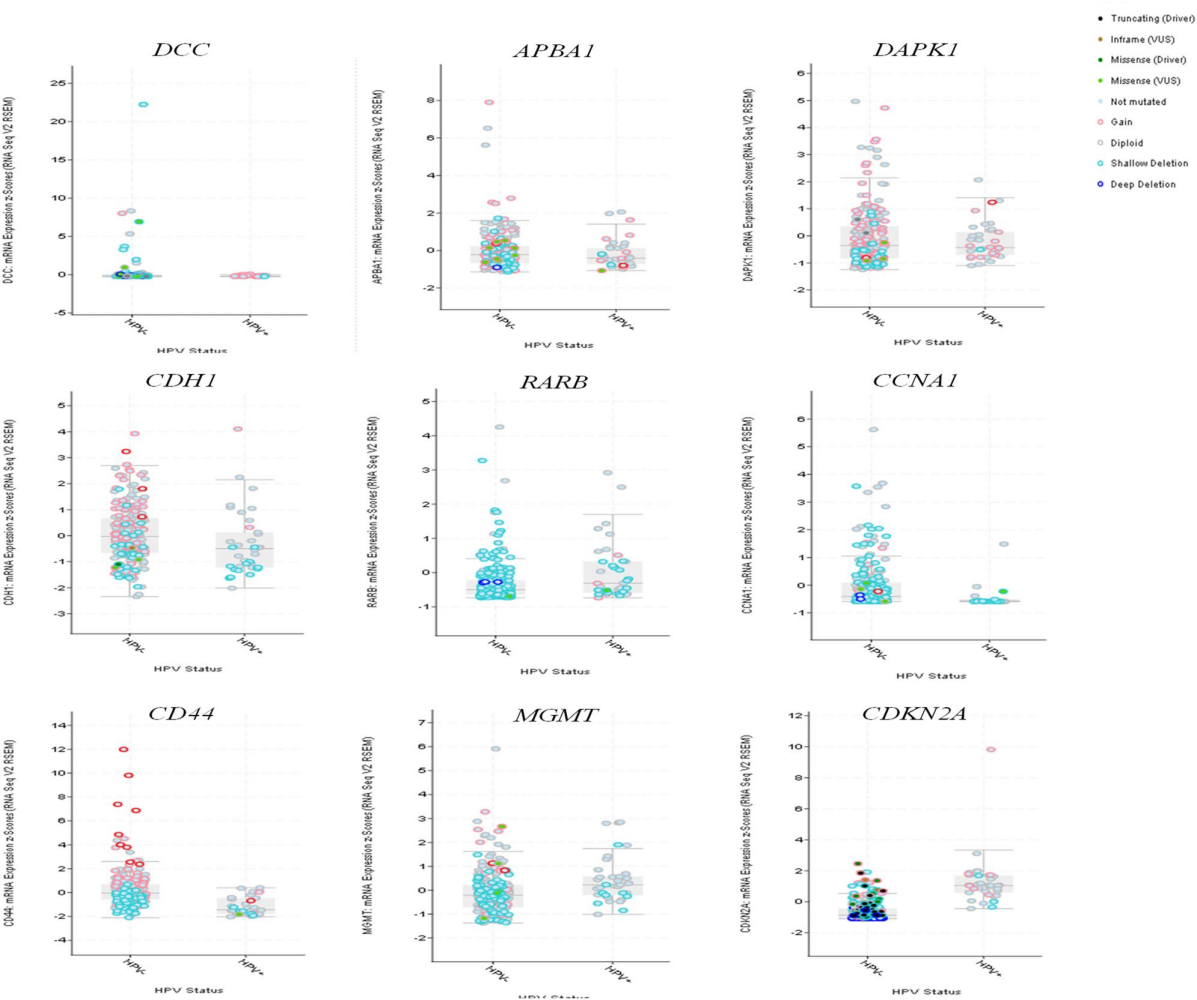
Validation of the gene expression in a large cohort of 279 HNC cases from Cancer Genome Atlas containing HM450 methylation, RNAseq data as well as information regarding alcohol, tobacco, and HPV infection. *CD44*, *CCNA1*, *DCC* and *TIMP3* were hypermethylated in the HNC HPV-negative cases. Image done using the open-access resource for interactive exploration of multidimensional cancer genomics data sets cBio Cancer Genomics Portal (http://cbioportal.org).

**Figure 4:**
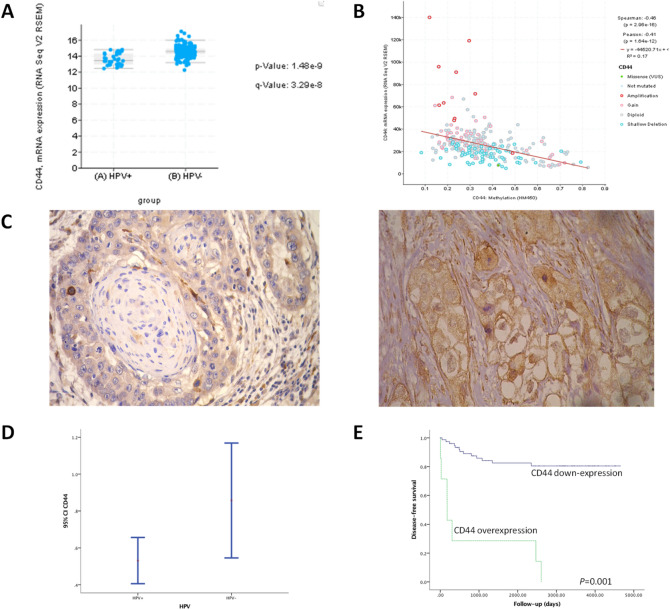
(**A**) Transcription profile reveals CD44 is highly expressed in HPV-HNC. (**B**) Correlation between gene expression and epigenetic alteration. (**C**) Validation of the protein expression in an independent cohort of 100 HNC with long-term follow-up. Representative immunohistochemical staining for CD44 in head and neck cancer. The cytoplasmic membrane immunoreactivity for CD44 was clearly identified. Original magnification: 400×. (**D**) CD44 protein was differentially expressed HPV+ and HPV- HNC patients. Confidence intervals (CI 95%) show relative percentage and IHC intensity value. Y-axis represents numerical values corresponding to the percentage and intensity of expression. (**E**) Survival curves analysis according to the Kaplan–Meier method showing that patients with positive expression of CD44 had shorter survival rate in comparison with negative immunostaining (log-rank test, *P* < 0.01).

Finally, to confirm if these genes associated with risk factors (alcohol, tobacco and HPV) might have impact on patient’s survival probability, we validated them using an independent large cohort of 279 HNC patients with high-throughput information from Cancer Genome Atlas containing HM450 methylation and RNAseq data^104^. For these analyses, we used tools available in the cBioPortal^105,106^. Not all these genes were statistically associated with alcohol and tobacco in this cohort. However, regarding HPV status, *CD44*, *CCNA1*, *DCC* and *TIMP3* were hypermethylated in the HNC HPV-negative (Fig. 3). The correlation between DNA hypermethylation and RNAseq data in this cohort confirms that DNA hypermethylation often leads to gene downregulation (Supplementary Fig. 1). There were no transcriptome data for *DCC* and *CCNA1* in this study^104^. For the eight genes that had transcriptome data available in the dataset, except for *APBA1,* we validated the negative correlation between DNA methylation (HM450 methylation platform) and gene expression (using RNAseq data). *CDH1* and *CD44* gene expression were significantly expressed in the HPV-positive patients (Fig. 4A,B). The methylation status (or any other alteration) of these genes alone did not achieve statistical significance on their impact for the overall survival based on this dataset, which included a mixed of different anatomical location and heterogenous tumor stage and histological grade.

In order to analyze whether this alteration affected the translational level, we explored these two promising candidates (*CD44* and *CDH1)* and their potential clinical impact by evaluating a cohort of 100 patients with unique tumor location at the tongue followed-up by 10 years (Fig. 4; Supplementary Fig. 1 and Supplementary Table 1). Typically, HNC patients relapse within 2 years. Among our studied patients, 23 (23.0%) had recurrence, 28 (28.0%) had distant metastasis, and 50 (50.0%) died. Sixty-nine patients from 85 HNC cases presenting negative staining for CD44 protein expression, had statistically better disease-free survival probability compared with patients whose tumors overexpressed CD44 (log-rank test, *P* < 0.01) (Fig. 4C-E). The lower expression of CD44 might reflect the reduced number of cells with stem cell properties which explain the absence of metastasis and the better survival rates.

### Prediction of the drugs to target the hypermethylated candidate genes

**Figure 5:**
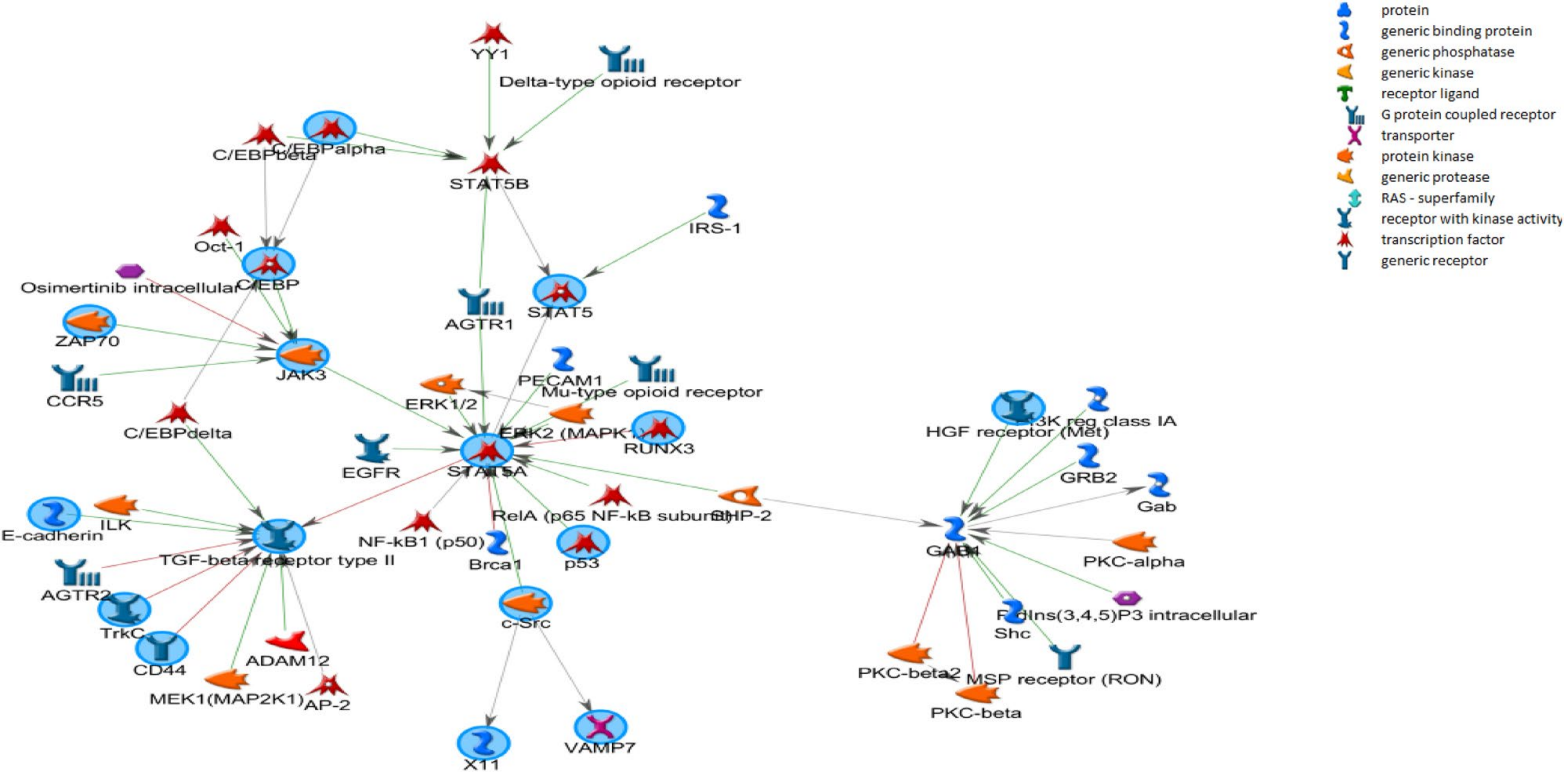
Regulatory network of selected hypermethylated genes associated with risk factors in head and neck cancer. Genes regulated by methylation from independent published studies in head and neck cancer (such as *GAB1*, *TGFB*, and *JAK3*) belongs to similar networks known to play a fundamental role in cancer progression. These methylated genes are targeted by the drugs, including TGF-beta receptor type II (Lerdelimumab, Suramin, and Interferon beta), GAB1-RA (Primidone, Flumazenil, Oxazepam, Flurazepam, Methylphenobarbital, Clorazepate, Ganaxolone, Clomethiazole, Zaleplon, Ocinaplon, Methyprylon, Indiplon, Zolpidem, Pentobarbital and Secobarbital), JAK 3 (Tofacitinib). Graphs were extracted from Metacore, Thompson Reuters (https://portal.genego.com).

To elucidate the underlying mechanisms of the hypermethylated genes in relation to the HNC susceptibility, these 120 known genes were used as seed for network growth. We identified six core biological processes (FDR < 10^–30^ and Z-score > ^90^), which were enriched for cell cycle regulation and metabolic pathways. Finally, based on this criteria, 53 methylated genes showed strong correlation with cancer risk, then, we searched for drugs interfering with these networks. We found 71 drugs targeting 18 proteins in the six networks identified (Supplementary Table 4). Proteins targeted by the drugs include TGF-beta receptor type II (Lerdelimumab, Suramin, and Interferon beta), GAB1-RA (Primidone, Flumazenil, Oxazepam, Flurazepam, Methylphenobarbital, Clorazepate, Ganaxolone, Clomethiazole, Zaleplon, Ocinaplon, Methyprylon, Indiplon, Zolpidem, Pentobarbital and Secobarbital), JAK 3 (Tofacitinib) (Fig. 5), IL-6 (Dexamethasone, Aloperine), CCND1 (Silibinin) and SRC (Cediranib, Nintedanib, Dasatinib/BMS-354825 and Saracatinib). The complete list of potential drugs acting on proteins associated with gene hypermethylation in head and neck cancer and their functions are presented in Supplementary Table 4.

## Discussion

In this systematic review we discussed and validated common genes regulated by DNA hypermethylation with fundamental role in HNC progression and metastatic competence, considering independent investigations with different HNC cohorts around the world. The clinical impact of these genes as prognostic factor is highly relevant to open-up new avenues to the therapeutic approach towards a personalized medicine. Although numerous advances in diagnosis and treatment have been achieved in the last years, 66% of HNC are still diagnosed at advanced stages (III or IV)^107^, 20% of the patients will develop an upper aerodigestive tract secondary tumor^2,19,109^ and more than 50% will died during the 5 years of follow-up due to the metastatic tumors.

The accumulation of epigenetic and genetic modifications, frequently associated with exposure to carcinogens, confer advantages to the cell in cancer division and survival, such as growth factor-independent proliferation, resistance to apoptosis, and an enhanced motility capability to migrate through the extracellular matrix (ECM) and invade adjacent tissues^110^. DNA methylation events is a critical tumor-specific event occurring early in tumor progression to metastasis and it can be easily detected by PCR in a manner that is minimally invasive to the patient^109^. Our review identified DNA methylation in 120 genes associated with high risk for developing HNC. The expression patterns of these hypermethylated genes were correlated with the risk factors and their impact for patient’s survival probability, indicating they can act as predictors in progressive HNC.

The multivariate analysis showed that numerous suppressor genes were significantly hypermethylated such as *P16*, *TIMP3, DCC, DAPK, MINT31, RARβ, MGMT, CCNA1, CD44,* and *CDH1*; these genes are involved in cell–cell adhesion, cell polarity and tissue morphogenesis. This gene was analyzed alone or in gene panels, however, the studies showed discordant results. In one report, *P16* hypermethylation was associated with carcinogenesis of oral epithelial dysplasia and it was considered a potential biomarker for the prediction of tumor progression of mild or moderate oral dysplasia^64,83^. The hypermethylation of the *P16* promoter gene has also been described in advanced oral cancer associated with increased risk of loco-regional recurrences^66^. Different degrees of *P16* hypermethylation have been reported in oral cancer^23,26,46,62,74,75,91,94^ and in others HNC location^73,93^.

Interestingly, promoter hypermethylation profile of the *P16, MGMT, GSTP1* and *DAPK* can be used as molecular biomarkers to detect recurrent tumors using liquid biopsy^111^. Since gene hypermethylation has been found to be a common and early event in several types of cancer, including HNC, it has emerged as a promising target for non-invasive detection strategies for tumor recurrence and metastasis. It was known that cancer cells shed their DNA into the bloodstream and that circulating free DNA (cfDNA) share molecular similarities with the primary tumor, including DNA hypermethylation. So, it has been suggested that tumor specific DNA hypermethylation in serum is useful for diagnosis and prediction prognosis^112^. This information is yet to be translated into useful and reliable tools for HNC in the clinical practice. Nonetheless, due to the increase of the sensitivity and the high-throughput quantitative methodologies for hypermethylation analysis, specific candidates will surely emerge by combination of different genetic and epigenetic panels to achieve accuracy in the neoplastic detection^113^. Over the next years, clinical trials on diagnostic and treatment approaches based on hypermethylation markers will be available for the assessment of HNC prognosis, therapeutic strategies and to predict the response to the treatment.

Researchers found significant differences in the tumorigenesis and HNC prognosis of patients with HPV-related cancer *versus* HPV-negative tumors and have tended to classify HPV associated malignancies as a distinct biologic entity. HPV-negative HNC is related to oral sexual behaviour, which is associated with HPV transmission^114,115^. Relative to HPV-negative malignancies, HPV-positive cancers are associated with a more favourable prognosis^114–116^. However, most patients (> 75%) with HPV-unassociated HNCs present tumors with poorer clinical outcome, do not respond to standard treatments due to a higher rate of relapses^115,116^. The majority of the studies included in our analysis, including HPV-positive patients, have strong association with alcohol and tobacco consumption. Previous studies suggested that although HPV-positive cancers in heavy smokers may be initiated through virus-related mutations, they go on to acquire tobacco-related mutations and become less dependent on the E6/E7 carcinogenesis mechanisms typically associated with the virus^117^. If epigenetic alteration can be modified by alcohol and tobacco status in HPV-positive patients, the gene silencing by hypermethylation can also be influenced by the combination of different risk factors, interfering not only in the tumor initiation process but also in the HNC progression to metastasis. A current limitation in the prognosis and therapeutic strategies of HNC is the lack of consistent methods and the use of large cohort studies to adequately address the influence of the etiologic complexity and the tumor heterogeneity (anatomical and histological) in the metastatic competence of this disease.

In this study, we firstly performed a systematic review to disclose potential candidates associated with HNC susceptibility that was confirmed by a validation in public platform from the TCGA datasets with 279 HNC cases. However, we also conducted an additional validation of the most relevant hypermethylated genes that showed statistical significance in both previous analysis by using an independent cohort with single tumor anatomical location (only tongue cancer) considering alcohol consumption, tobacco use and HPV status. After this screening, only CD44 expression showed significant clinical impact at the translational level being associated with tumor recurrence. CD44 is a well-characterized cell surface glycoprotein receptor associated with a subpopulation of resilient tumor cells with enhanced carcinogenic properties specially involved with increased cell migration. We confirmed the increased proportions of CD44 + cells correlated with poor patient’s outcome in HPV negative HNC patients. The lower expression of CD44 might reflect the reduced number of cells with stem cell properties which explain the absence of metastasis and the better survival rates. In HNC, CD44 + expression has been associated with tumor-initiating cells or cancer stem cells due to their ability to persist and self-renew following therapy. Extensive investigations in our field have been performed with a hope to find a new prognostic tool to understand the basis of molecular carcinogenesis in HNC but also to identify potential therapeutic opportunities toward personalized medicine to manage patients with advanced disease. The ability to manipulate DNA methylation status and gene function by local and systemic delivery of epigenetic drugs (methylation inhibitors [e.g., 5-azacytidine]; antisense oligonucleotides [e.g., MG98]; and small molecule DNA methylation inhibitor [RG108]) has recently gained interest as novel therapeutic approach. Here, we reported potential drugs to target the most common alteration proposed in literature related to DNA hypermethylation in progressive HNC. The list of drugs available (Supplementary Table 4) may be used to block multiple nodes in critical pathways involved in cell proliferation, differentiation, tumor growth and survival in HNC at high-risk for recurrence.

In summary, this review highlights the impact of DNA hypermethylation associated with the main risk factors for HNC and show, from independent studies, the implication of methylated genes in the regulation of critical network with fundamental role in cancer progression to metastasis, which could be used as a potential therapeutic target and long-term surveillance for patients with invasive HNC.

## Supplementary Information

Supplementary Information 1.

Supplementary Information 2.
