# Supplementary material for: Portrait of DNA methylated genes predictive of poor prognosis in head and neck cancer and the implication for targeted therapy
Source: Sci Rep. 2021 May 11;11:10012. doi: 10.1038/s41598-021-89476-x (PMC8113272; doi:10.1038/s41598-021-89476-x)
Supplement: Supplementary file 1 — Supplementary Information 1. [file 41598_2021_89476_MOESM1_ESM.pdf]

Supplementary Figure 1

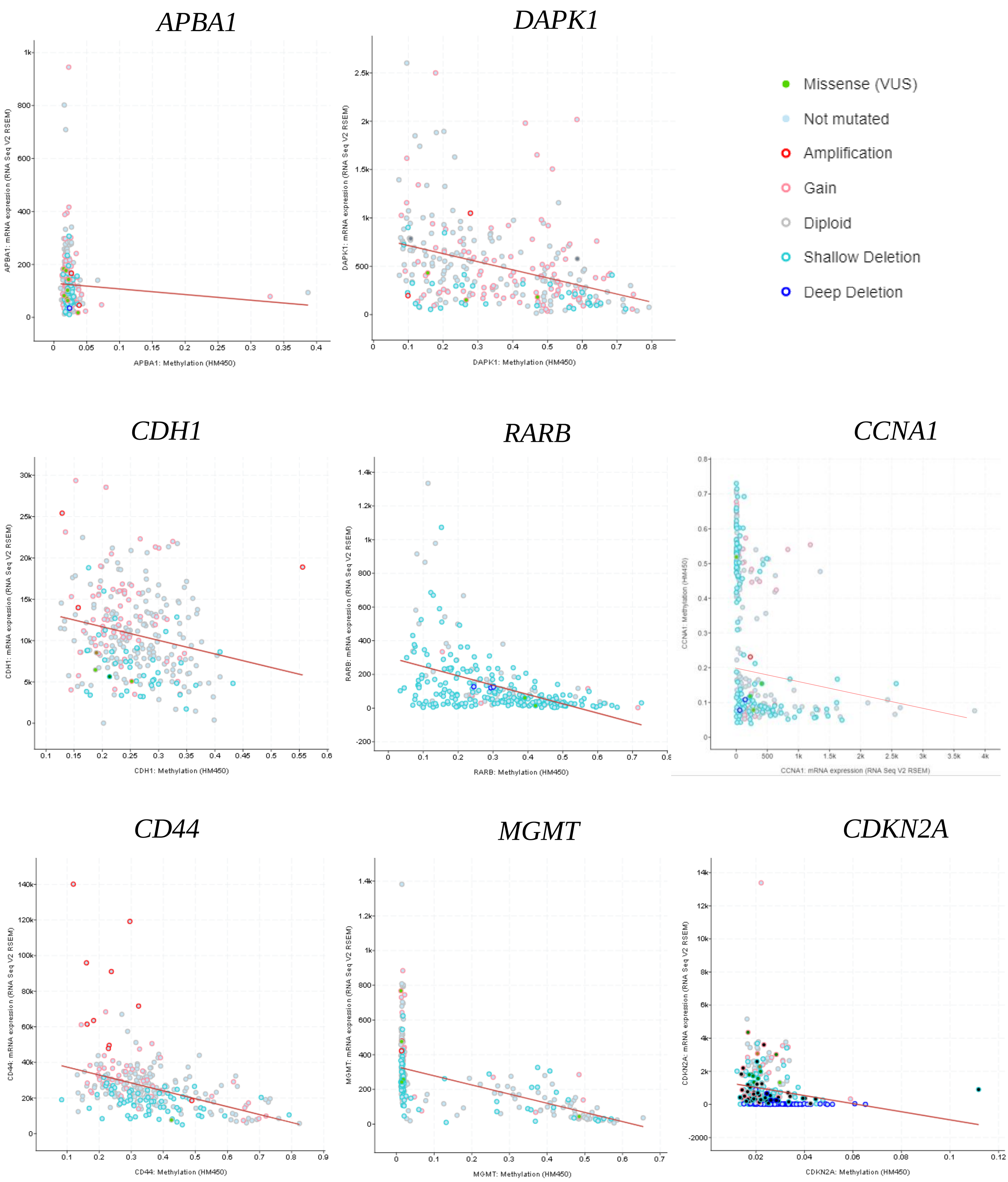

Supplementary Figure 1: The correlation between DNA hypermethylation and RNAseq data using an independent large cohort of 279 HNC patients with high-throughput information from Cancer Genome Atlas confirms that DNA hypermethylation often leads to gene downregulation.

Supplementary Figure 2

CDH1

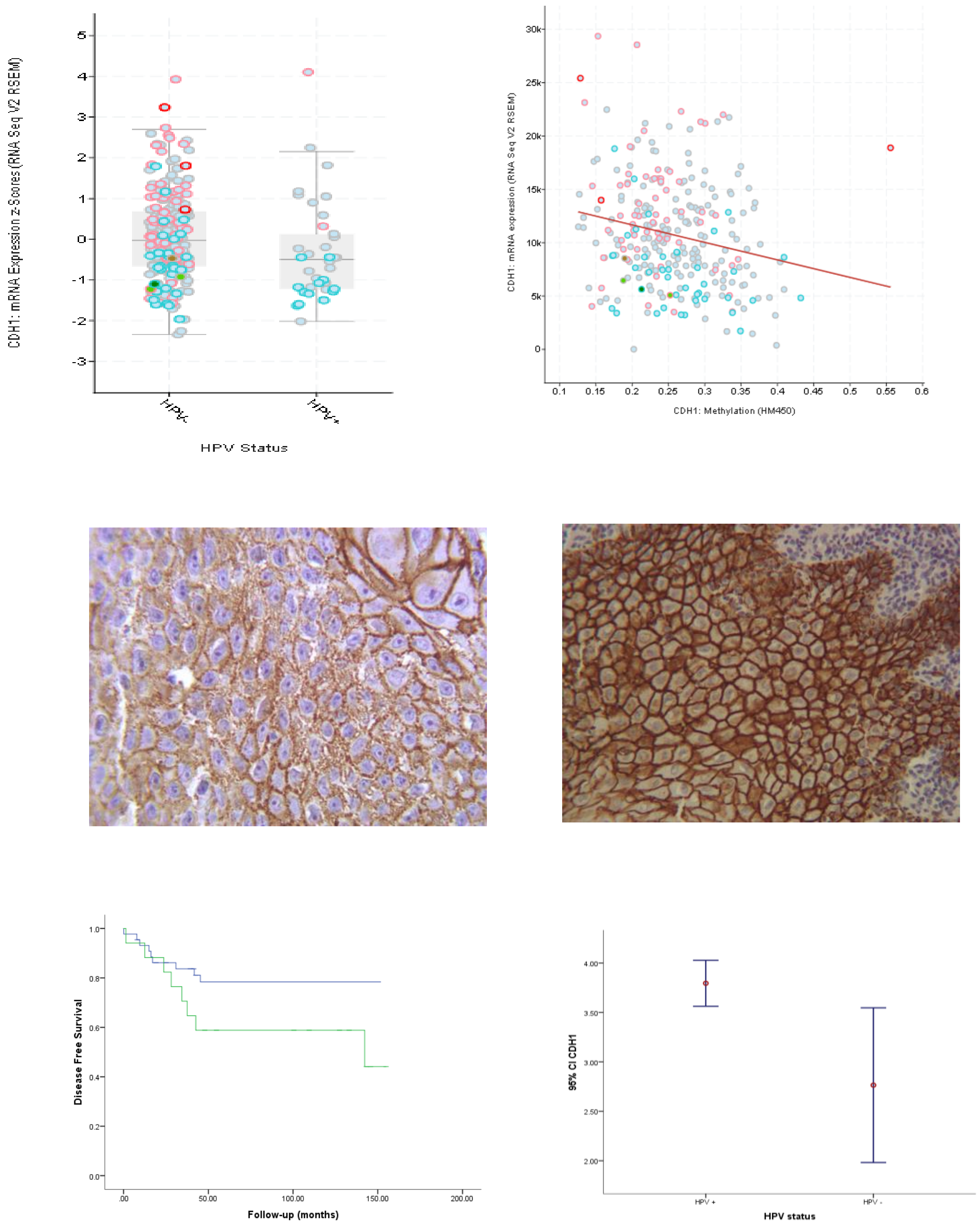

Supplementary Figure 2: – (A) Transcription profile reveals CDH1 is highly expressed in HPV-HNC. (B) Correlation between gene expression and epigenetic alteration. (C) Validation of the protein expression in an independent cohort of 100 HNC with long-term follow-up. Representative immunohistochemical staining for CDH1 in head and neck cancer. The cytoplasmic membrane immunoreactivity for CDH1 was clearly identified. Original magnification: 400x. (D) CD44 protein was differentially expressed HPV+ and HPV- HNC patients. Confidence intervals (CI: 95%) show relative percentage and IHC intensity value. Y-axis represents numerical values corresponding to the percentage and intensity of expression. (E) Survival curves analysis according to the Kaplan–Meier method showing that patients with positive expression of CD44 had shorter survival rate in comparison with negative immunostaining (log-rank test, P<0.01).
