# Supplementary material for: Portrait of DNA methylated genes predictive of poor prognosis in head and neck cancer and the implication for targeted therapy
Source: Sci Rep. 2021 May 11;11:10012. doi: 10.1038/s41598-021-89476-x (PMC8113272; doi:10.1038/s41598-021-89476-x)
Supplement: Supplementary file 2 — Supplementary Information 2. [file 41598_2021_89476_MOESM2_ESM.docx]

**PORTRAIT OF DNA METHYLATED GENES PREDICTIVE OF POOR PROGNOSIS IN HEAD AND NECK CANCER AND THE IMPLICATION FOR TARGETED THERAPY**

Jessica Hier^1^, Olivia Vachon^1^, Allison Bernstein^1^, Iman Ibrahim^1^, Alex Mlynarek^1^, Michael Hier^1^, Moulay A. Alaoui-Jamali^2^, Mariana Maschietto^3^, Sabrina Daniela da Silva^1,2*^

***Corresponding author**

Sabrina Daniela da Silva

*sabrina.wurzba@gmail.com*

Department of Otolaryngology-Head and Neck Surgery - McGill University

Lady Davis Institute for Medical Research and Segal Cancer Centre

Sir Mortimer B. Davis-Jewish General Hospital

3755 Côte Ste-Catherine Road – Montreal (QC)

Canada, H3T 1E2

**Supplementary Table 1:** Clinicopathological characteristics of 100 patients with head and neck cancer.

| **Variable** | **Category** | **Paraffin-embedded samples *n* (%)** | |
| --- | --- | --- | --- |
|  |  | **Non-metastatic** | **Metastatic** |
| Age | < 55 years old | 39 (54.2) | 15 (53.6) |
|  | ≥ 55 years old | 33 (45.8) | 13 (46.4) |
| Gender | Male | 56 (77.8) | 25 (89.3) |
|  | Female | 16 (22.2) | 3 (10.7) |
| Smoking habit | No | 7 (10.8) | 1 (5) |
|  | Yes | 58 (89.2) | 19 (95) |
| Alcohol consumption | No | 16 (24.6) | 2 (10) |
|  | Yes | 49 (75.4) | 18 (90) |
| *T category | T1 + T2 | 28 (39.4) | 5 (22.7) |
|  | T3 + T4 | 43 (60.6) | 17 (77.3) |
| *N category | N0 | 45 (63.4) | 1 (4.3) |
|  | N+ | 26 (36.6) | 22 (95.7) |
| Recurrence or metastasis | No | 72 (100) | 0 |
|  | Yes | 0 | 28 (100) |
| Death | No | 43 (59.7) | 6 (21.4) |
|  | Yes | 29 (40.3) | 22 (78.6) |

*T: tumor size; N: lymph nodes involvement

**Supplementary Table 2:** Enriched genes for biological processes related to cell proliferation and death, response to stimulus (including drugs), metabolism, and cellular motility and differentiation in head and neck cancer.

| **Gene Set Name [# Genes (K)]** | **Description** | **# Genes in Overlap (k)** | **p-value** | **FDR q-value** |
| --- | --- | --- | --- | --- |
| GO_REGULATION_OF_CELL_POPULATION_PROLIFERATION [1716] | Any process that modulates the frequency, rate or extent of cell proliferation. [GOC:jl] | 36 | 1.51 e-22 | 1.13 e-18 |
| GO_APOPTOTIC_SIGNALING_PATHWAY [603] | A series of molecular signals which triggers the apoptotic death of a cell. The pathway starts with reception of a signal and ends when the execution phase of apoptosis is triggered. [GOC:mtg_apoptosis] | 23 | 5.62 e-20 | 2.12 e-16 |
| GO_RESPONSE_TO_ENDOGENOUS_STIMULUS [1721] | Any process that results in a change in state or activity of a cell or an organism (in terms of movement, secretion, enzyme production, gene expression, etc.) as a result of a stimulus arising within the organism. [GOC:sm] | 33 | 2.06 e-19 | 5.16 e-16 |
| GO_NEGATIVE_REGULATION_OF_CELL_POPULATION_PROLIFERATION [763] | Any process that stops, prevents or reduces the rate or extent of cell proliferation. [GOC:go_curators] | 24 | 6.63 e-19 | 1.25 e-15 |
| GO_CELLULAR_RESPONSE_TO_ENDOGENOUS_STIMULUS (1454) | Any process that results in a change in state or activity of a cell (in terms of movement, secretion, enzyme production, gene expression, etc.) as a result of a stimulus arising within the organism. [GOC:mah] | 30 | 1.76 e-18 | 2.34 e-15 |
| GO_PROTEIN_PHOSPHORYLATION [1994] | The process of introducing a phosphate group on to a protein. [GOC:hb] | 34 | 1.87 e-18 | 2.34 e-15 |
| GO_REGULATION_OF_CELL_DEATH [1748] | Any process that modulates the rate or frequency of cell death. Cell death is the specific activation or halting of processes within a cell so that its vital functions markedly cease, rather than simply deteriorating gradually over time, which culminates in cell death. [GOC:dph, GOC:tb] | 32 | 3.18 e-18 | 3.42 e-15 |
| GO_CELL_MOTILITY [1758] | Any process involved in the controlled self-propelled movement of a cell that results in translocation of the cell from one place to another. [GOC:dgh, GOC:dph, GOC:isa_complete, GOC:mlg] | 32 | 3.75 e-18 | 3.53 e-15 |
| GO_NEGATIVE_REGULATION_OF_SIGNALING [1427] | Any process that stops, prevents, or reduces the frequency, rate or extent of a signaling process. [GOC:mtg_signal] | 29 | 1.1 e-17 | 8.77 e-15 |
| GO_NEGATIVE_REGULATION_OF_PROTEIN_METABOLIC_PROCESS [1189] | Any process that stops, prevents, or reduces the frequency, rate or extent of chemical reactions and pathways involving a protein. [GOC:ai] | 27 | 1.17 e-17 | 8.77 e-15 |
| GO_NEGATIVE_REGULATION_OF_RESPONSE_TO_STIMULUS [1703] | Any process that stops, prevents, or reduces the frequency, rate or extent of a response to a stimulus. Response to stimulus is a change in state or activity of a cell or an organism (in terms of movement, secretion, enzyme production, gene expression, etc.) as a result of a stimulus. [GOC:jid] | 31 | 1.42 e-17 | 9.72 e-15 |
| GO_REGULATION_OF_RESPONSE_TO_STRESS [1559] | Any process that modulates the frequency, rate or extent of a response to stress. Response to stress is a change in state or activity of a cell or an organism (in terms of movement, secretion, enzyme production, gene expression, etc.) as a result of a disturbance in organismal or cellular homeostasis, usually, but not necessarily, exogenous (e.g. temperature, humidity, ionizing radiation). [GOC:dhl] | 29 | 1.13 e-16 | 6.95 e-14 |
| GO_LOCOMOTION [1984] | Self-propelled movement of a cell or organism from one location to another. [GOC:dgh] | 32 | 1.2 e-16 | 6.95 e-14 |
| GO_REGULATION_OF_CELLULAR_COMPONENT_MOVEMENT [1107] | Any process that modulates the frequency, rate or extent of the movement of a cellular component. [GOC:ai, GOC:dph, GOC:jl] | 25 | 2.59 e-16 | 1.39 e-13 |
| GO_REGULATION_OF_PHOSPHORYLATION [1625] | Any process that modulates the frequency, rate or extent of addition of phosphate groups into a molecule. [GOC:jl] | 29 | 3.33 e-16 | 1.67 e-13 |
| GO_NEGATIVE_REGULATION_OF_PHOSPHORYLATION [469] | Any process that stops, prevents or decreases the rate of addition of phosphate groups to a molecule. [GOC:jl] | 18 | 7.97 e-16 | 3.75 e-13 |
| GO_POSITIVE_REGULATION_OF_CELL_DEATH [737] | Any process that increases the rate or frequency of cell death. Cell death is the specific activation or halting of processes within a cell so that its vital functions markedly cease, rather than simply deteriorating gradually over time, which culminates in cell death. [GOC:dph, GOC:tb] | 21 | 9.39 e-16 | 4.09 e-13 |
| GO_NEGATIVE_REGULATION_OF_MOLECULAR_FUNCTION [1174] | Any process that stops or reduces the rate or extent of a molecular function, an elemental biological activity occurring at the molecular level, such as catalysis or binding. [GO:jl] | 25 | 9.97 e-16 | 4.09 e-13 |
| GO_POSITIVE_REGULATION_OF_DEVELOPMENTANTAL_PROCESS [1428] | Any process that activates or increases the rate or extent of development, the biological process whose specific outcome is the progression of an organism over time from an initial condition (e.g. a zygote, or a young adult) to a later condition (e.g. a multicellular animal or an aged adult). [GOC:ai] | 27 | 1.06 e-15 | 4.09 e-13 |
| GO_RESPONSE_TO_DRUG [1061] | Any process that results in a change in state or activity of a cell or an organism (in terms of movement, secretion, enzyme production, gene expression, etc.) as a result of a drug stimulus. A drug is a substance used in the diagnosis, treatment or prevention of a disease. [GOC:jl] | 24 | 1.09 e-15 | 4.09 e-13 |
| GO_POSITIVE_REGULATION_OF_NUCLEOBASE_CONTAINING_COMPOUND_METABOLIC_PROCESS [1870] | Any cellular process that activates or increases the frequency, rate or extent of the chemical reactions and pathways involving nucleobases, nucleosides, nucleotides and nucleic acids. [GOC:go_curators] | 30 | 1.57 e-15 | 5.65 e-13 |
| GO_REGULATION_OF_PROTEIN_MODIFICATION_PROCESS [1880] | Any process that modulates the frequency, rate or extent of the covalent alteration of one or more amino acid residues within a protein. [GOC:mah, GOC:tb] | 30 | 1.81 e-15 | 6.21 e-13 |
| GO_NEGATIVE_REGULATION_OF_TRANSFERASE_ACTIVITY [286] | Any process that stops or reduces the rate of transferase activity, the catalysis of the transfer of a group, e.g. a methyl group, glycosyl group, acyl group, phosphorus-containing, or other groups, from a donor compound to an acceptor. [GOC:ai] | 15 | 2.54 e-15 | 8.32 e-13 |
| GO_GLAND_DEVELOPMENT [437] | The process whose specific outcome is the progression of a gland over time, from its formation to the mature structure. A gland is an organ specialised for secretion. [GOC:jid] | 17 | 4.33 e-15 | 1.33 e-12 |
| GO_REGULATION_OF_PROTEIN_SERINE_THREONINE-KINASE_ACTIVITY [518] | Any process that modulates the rate, frequency, or extent of protein serine/threonine kinase activity. [GOC:mah] | 18 | 4.43 e-15 | 1.33 e-12 |
| GO_NEGATIVE_REGULATION_OF_BIOSYNTHETIC_PROCESS [1673] | Any process that stops, prevents, or reduces the rate of the chemical reactions and pathways resulting in the formation of substances. [GOC:go_curators] | 28 | 5.98 e-15 | 1.73 e-12 |
| GO_REGULATION_OF_PHOSPHORUS_METABOLIC_PROCESS [1823] | Any process that modulates the frequency, rate or extent of the chemical reactions and pathways involving phosphorus or compounds containing phosphorus. [GOC:ai] | 29 | 6.47 e-15 | 1.8 e-12 |
| GO_POSITIVE_REGULATION_OF_CELLULAR_BIOSYNTHETIC_PROCESS [1988] | Any process that activates or increases the frequency, rate or extent of the chemical reactions and pathways resulting in the formation of substances, carried out by individual cells. [GOC:mah] | 30 | 7.93 e-15 | 2.08 e-12 |
| GO_NEGATIVE_REGULATION_OF_CATALYTIC_ACTIVITY [823] | Any process that stops or reduces the activity of an enzyme. [GOC:ebc, GOC:jl, GOC:tb, GOC:vw] | 21 | 8.18 e-15 | 2.08 e-12 |
| GO_DIGESTIVE_SYSTEM_DEVELOPMENT [146] | The process whose specific outcome is the progression of the digestive system over time, from its formation to the mature structure. The digestive system is the entire structure in which digestion takes place. Digestion is all of the physical, chemical, and biochemical processes carried out by multicellular organisms to break down ingested nutrients into components that may be easily absorbed and directed into metabolism. [GOC:jid] | 12 | 8.29 e-15 | 2.08 e-12 |
| GO_POSITIVE_REGULATION_OF_RNA_METABOLIC_PROCESS [1710] | Any process that activates or increases the frequency, rate or extent of the chemical reactions and pathways involving RNA. [GOC:ai] | 28 | 1.03 e-14 | 2.5 e-12 |
| GO_RESPONSE_TO_ORGANIC_CYCLIC_COMPOUND [973] | Any process that results in a change in state or activity of a cell or an organism (in terms of movement, secretion, enzyme production, gene expression, etc.) as a result of an organic cyclic compound stimulus. [GOC:ef] | 22 | 2.02 e-14 | 4.74 e-12 |
| GO_TUBE_DEVELOPMENT [1122] | The process whose specific outcome is the progression of a tube over time, from its initial formation to a mature structure. Epithelial and endothelial tubes transport gases, liquids and cells from one site to another and form the basic structure of many organs and tissues including lung and trachea, kidney, the mammary gland, the vascular system and the gastrointestinal and urinary-genital tracts. [PMID:12526790] | 23 | 3.7 e-14 | 8.3 e-12 |
| GO_NEGATIVE_REGULATION_OF_PHOSPHORUS_METABOLIC_PROCESS [587] | Any process that decreases the frequency, rate or extent of the chemical reactions and pathways involving phosphorus or compounds containing phosphorus. [GOC:dph, GOC:tb] | 18 | 3.75 e-14 | 8.3 e-12 |
| GO_EXTRINSIC_APOPTOTIC_SIGNALING_PATHWAY [223] | A series of molecular signals in which a signal is conveyed from the cell surface to trigger the apoptotic death of a cell. The pathway starts with either a ligand binding to a cell surface receptor, or a ligand being withdrawn from a cell surface receptor (e.g. in the case of signaling by dependence receptors), and ends when the execution phase of apoptosis is triggered. [GOC:mtg_apoptosis, GOC:yaf, PMID:17340152] | 13 | 5.23 e-14 | 1.13 e-11 |
| GO_EPITHELIUM_DEVELOPMENT [1296] | The process whose specific outcome is the progression of an epithelium over time, from its formation to the mature structure. An epithelium is a tissue that covers the internal or external surfaces of an anatomical structure. [GOC:dph, GOC:mtg_lung] | 24 | 8.5 e-14 | 1.78 e-11 |
| GO_NEGATIVE_REGULATION_OF_DEVELOPMENTAL_PROCESS [1047] | Any process that stops, prevents or reduces the rate or extent of development, the biological process whose specific outcome is the progression of an organism over time from an initial condition (e.g. a zygote, or a young adult) to a later condition (e.g. a multicellular animal or an aged adult). [GOC:ai] | 22 | 8.79 e-14 | 1.79 e-11 |
| GO_NEGATIVE_REGULATION_OF_PROTEIN_MODI | Any process that stops, prevents, or reduces the frequency, rate or extent of the covalent alteration of one or more amino acid residues within a protein. [GOC:mah, GOC:tb] | 18 | 9.47 e-14 | 1.88 e-11 |
| ODIFICATION_PROCESS [620] |  |  |  |  |
| GO_REGULATION_OF_CELL_DIFFERENTIATION [1881] | Any process that modulates the frequency, rate or extent of cell differentiation, the process in which relatively unspecialized cells acquire specialized structural and functional features. [GOC:go_curators] | 28 | 1.07 e-13 | 2.07 e-11 |
| GO_RESPONSE_TO_GROWTH_FACTOR [732] | Any process that results in a change in state or activity of a cell or an organism (in terms of movement, secretion, enzyme production, gene expression, etc.) as a result of a growth factor stimulus. [GOC:BHF, GOC:mah] | 19 | 1.33 e-13 | 2.5 e-11 |
| GO_NEGATIVE_REGULATION_OF_NUCLEOBASE_CONTAINING_COMPOUND_METABOLIC_PROCESS [1470] | Any cellular process that stops, prevents, or reduces the frequency, rate or extent of the chemical reactions and pathways involving nucleobases, nucleosides, nucleotides and nucleic acids. [GOC:go_curators] | 25 | 1.57 e-13 | 2.89 e-11 |
| GO_NEUROGENESIS [1625] | Generation of cells within the nervous system. [GO_REF:0000021, GOC:cls, GOC:curators, GOC:dgh, GOC:dph, GOC:jid, GOC:mtg_15jun06] | 26 | 1.9 e-13 | 3.41 e-11 |
| GO_REGULATION_OF_TRANSFERASE_ACTIVITY [976] | Any process that modulates the frequency, rate or extent of transferase activity, the catalysis of the transfer of a group, e.g. a methyl group, glycosyl group, acyl group, phosphorus-containing, or other groups, from one compound (generally regarded as the donor) to another compound (generally regarded as the acceptor). Transferase is the systematic name for any enzyme of EC class 2. [EC:2.-.-.-, GOC:ai] | 21 | 2.21 e-13 | 3.87 e-11 |
| GO_REGULATION_OF_KINASE_ACTIVITY [872] | Any process that modulates the frequency, rate or extent of kinase activity, the catalysis of the transfer of a phosphate group, usually from ATP, to a substrate molecule. [GOC:bf] | 20 | 2.75 e-13 | 4.65 e-11 |
| GO_NEGATIVE_REGULATION_OF_PROTEIN_SERINE_THREONINE_KINASE_ACTIVITY [145] | Any process that decreases the rate, frequency, or extent of protein serine/threonine kinase activity. [GOC:BHF, GOC:mah] | 11 | 2.78 e-13 | 4.65 e-11 |
| GO_NEGATIVE_REGULATION_OF_KINASE_ACTIVITY [258] | Any process that stops, prevents, or reduces the frequency, rate or extent of kinase activity, the catalysis of the transfer of a phosphate group, usually from ATP, to a substrate molecule. [GOC:mah] | 13 | 3.38 e-13 | 5.54 e-11 |
| GO_POSITIVE_REGULATION_OF_CELL_DIFFERENTIATION [1000] | Any process that activates or increases the frequency, rate or extent of cell differentiation. [GOC:go_curators] | 21 | 3.51 e-13 | 5.63 e-11 |
| GO_POSITIVE_REGULATION_OF_MULTICELLULAR_ORGANISMAL_PROCESS [1825] | Any process that activates or increases the frequency, rate or extent of an organismal process, any of the processes pertinent to the function of an organism above the cellular level; includes the integrated processes of tissues and organs. [GOC:ai] | 27 | 3.76 e-13 | 5.9 e-11 |
| GO_REGULATION_OF_APOPTOTIC_SIGNALING_PATHWAY [408] | Any process that modulates the frequency, rate or extent of apoptotic signaling pathway. [GOC:mtg_apoptosis] | 15 | 4.48 e-13 | 6.88 e-11 |
| GO_RESPONSE_TO_OXYGEN_CONTAINING_COMPOUND [1698] | Any process that results in a change in state or activity of a cell or an organism (in terms of movement, secretion, enzyme production, gene expression, etc.) as a result of an oxygen-containing compound stimulus. [GOC:pr, GOC:TermGenie] | 26 | 5.17 e-13 | 7.78 e-11 |

**Supplementary Table 3:** Enriched cellular pathways in head and neck cancer related with DNA hypermethylation.

| **Gene Set Name [# Genes (K)]** | **Description** | **# Genes in Overlap (k)** | **p-value** | **FDR q-value** |
| --- | --- | --- | --- | --- |
| KEGG_PATHWAYS_IN_CANCER [325] | Pathways in cancer | 18 | 1.27 e-18 | 2.37 e-16 |
| KEGG_BLADDER_CANCER [42] | Bladder cancer | 7 | 2.32 e-11 | 2.16 e-9 |
| KEGG_P53_SIGNALING_PATHWAY [68] | p53 signaling pathway | 7 | 7.88 e-10 | 4.89 e-8 |
| KEGG_MELANOMA [71] | Melanoma | 7 | 1.07 e-9 | 4.99 e-8 |
| KEGG_NON_SMALL_CELL_LUNG_CANCER [54] | Non-small cell lung cancer | 6 | 8.39 e-9 | 3.12 e-7 |
| KEGG_CELL_CYCLE [124] | Cell cycle | 7 | 5.4 e-8 | 1.67 e-6 |
| KEGG_ENDOMETRIAL_CANCER [52] | Endometrial cancer | 5 | 3.26 e-7 | 8.65 e-6 |
| KEGG_GLIOMA [65] | Glioma | 5 | 1.01 e-6 | 2.34 e-5 |
| KEGG_PANCREATIC_CANCER [70] | Pancreatic cancer | 5 | 1.46 e-6 | 3.01 e-5 |
| KEGG_CHRONIC_MYELOID_LEUKEMIA [73] | Chronic myeloid leukemia | 5 | 1.8 e-6 | 3.34 e-5 |
| KEGG_SMALL_CELL_LUNG_CANCER [84] | Small cell lung cancer | 5 | 3.61 e-6 | 5.83 e-5 |
| KEGG_WNT_SIGNALING_PATHWAY [150] | Wnt signaling pathway | 6 | 3.76 e-6 | 5.83 e-5 |
| KEGG_PROSTATE_CANCER [89] | Prostate cancer | 5 | 4.8 e-6 | 6.87 e-5 |
| KEGG_ACUTE_MYELOID_LEUKEMIA [57] | Acute myeloid leukemia | 4 | 1.86 e-5 | 2.48 e-4 |
| KEGG_COLORECTAL_CANCER [62] | Colorectal cancer | 4 | 2.6 e-5 | 3.23 e-4 |
| KEGG_FOCAL_ADHESION [199] | Focal adhesion | 5 | 2.25 e-4 | 2.62 e-3 |
| KEGG_MAPK_SIGNALING_PATHWAY [267] | MAPK signaling pathway | 5 | 8.58 e-4 | 9.32 e-3 |
| KEGG_NEUROACTIVE_LIGAND_RECEPTOR_INTERACTION [272] | Neuroactive ligand-receptor interaction | 5 | 9.32 e-4 | 9.32 e-3 |
| KEGG_METABOLISM_OF_XENOBIOTICS_BY_CYTOCHROME_P450 [70] | Metabolism of xenobiotics by cytochrome P450 | 3 | 9.52 e-4 | 9.32 e-3 |
| KEGG_DRUG_METABOLISM_CYTOCHROME_P450 [72] | Drug metabolism - cytochrome P450 | 3 | 1.03 e-3 | 9.52 e-3 |
| KEGG_ADHERENS_JUNCTION [73] | Adherens junction | 3 | 1.07 e-3 | 9.52 e-3 |
| KEGG_ECM_RECEPTOR_INTERACTION [84] | ECM-receptor interaction | 3 | 1.61 e-3 | 1.36 e-2 |
| KEGG_ERBB_SIGNALING_PATHWAY [87] | ErbB signaling pathway | 3 | 1.78 e-3 | 1.38 e-2 |
| KEGG_HEMATOPOIETIC_CELL_LINEAGE [87] | Hematopoietic cell lineage | 3 | 1.78 e-3 | 1.38 e-2 |
| KEGG_THYROID_CANCER [29] | Thyroid cancer | 2 | 2.86 e-3 | 2.13 e-2 |
| KEGG_PRIMARY_IMMUNODEFICIENCY [35] | Primary immunodeficiency | 2 | 4.15 e-3 | 2.97 e-2 |
| KEGG_NEUROTROPHIN_SIGNALING_PATHWAY [126] | Neurotrophin signaling pathway | 3 | 5.07 e-3 | 3.49 e-2 |
| KEGG_CYTOKINE_CYTOKINE_RECEPTOR_INTERACTION [265] | Cytokine-cytokine receptor interaction | 4 | 6.15 e-3 | 4.08 e-2 |

**Supplementary Table 4:** Potential drugs to target genes with DNA hypermethylation alteration in head and neck cancer.

| **Gene Symbol** | **Target** | **Drug** | **Effect** |
| --- | --- | --- | --- |
| IL6 | IL-6 | Dexamethasone extracellular region | Inhibition |
|  |  | Aloperine extracellular region | Inhibition |
| JAK3 | JAK3 | Tofacitinib intracellular | Inhibition |
| TGFBR2 | TGF-beta receptor type II | Suramin extracellular region | Inhibition |
| GABRA1 | GABA-A receptor alpha-1 subunit | Primidone extracellular region | Unspecified |
|  |  | Flumazenil intracellular | Unspecified |
|  |  | Clorazepate extracellular region | Activation |
|  |  | Oxazepam extracellular region | Activation |
|  |  | Methylphenobarbital extracellular region | Activation |
|  |  | Ganaxolone extracellular region | Activation |
|  |  | Clomethiazole extracellular region | Activation |
|  |  | Zaleplon extracellular region | Activation |
|  |  | Ocinaplon extracellular region | Activation |
|  |  | Methyprylon extracellular region | Activation |
|  |  | Indiplon intracellular | Activation |
|  |  | Zolpidem extracellular region | Activation |
|  |  | Pentobarbital extracellular region | Activation |
|  |  | Flurazepam extracellular region | Activation |
|  |  | Secobarbital extracellular region | Inhibition |
| EDNRB | EDNRB | Tezosentan extracellular region | Inhibition |
|  |  | Enrasentan extracellular region | Inhibition |
| RARB | RARbeta | Adapalene intracellular | Activation |
|  |  | Tazarotene intracellular | Activation |
|  |  | Tamibarotene intracellular | Activation |
|  |  | Retinoic acid intracellular | Activation |
|  |  | Etretinate intracellular | Activation |
| SRC | c-Src | Cediranib intracellular | Inhibition |
|  |  | Nintedanib intracellular | Inhibition |
|  |  | Dasatinib intracellular | Inhibition |
|  |  | Saracatinib intracellular | Inhibition |
| ABCB1 | MDR1 | Doxorubicin cytoplasm | Inhibition |
|  |  | Tamoxifen intracellular | Inhibition |
|  |  | Tariquidar extracellular region | Inhibition |
|  |  | Valspodar extracellular region | Inhibition |
| KCNQ1 | KCNQ1 | Indapamide extracellular region | Inhibition |
|  |  | Azimilide extracellular region | Inhibition |
| RARA | RARalpha | Tamibarotene intracellular | Activation |
|  |  | Retinoic acid intracellular | Activation |
|  |  | Isotretinoin intracellular | Unspecified |
|  |  | Amsilarotene intracellular | Unspecified |
|  |  | Etretinate intracellular | Activation |
| RB1 | Rb protein | Amsilarotene intracellular | Activation |
| TACR1 | Substance P receptor | AV608 extracellular region | Inhibition |
|  |  | TAK637 extracellular region | Inhibition |
|  |  | Aprepitant extracellular region | Inhibition |
|  |  | R673 extracellular region | Inhibition |
|  |  | Nolpitantium extracellular region | Inhibition |
| COL1A2 | COL1A2 | Halofuginone extracellular region | Inhibition |
| EPHA2 | Ephrin-A receptor 2 | Dasatinib intracellular | Inhibition |
| MET | HGF receptor (Met) | Glesatinib extracellular region | Inhibition |
|  |  | Cabozantinib intracellular | Inhibition |
|  |  | Foretinib extracellular region | Inhibition |
|  |  | Tivantinib extracellular region | Inhibition |
|  |  | Crizotinib intracellular | Inhibition |
| CDKN1B | p27KIP1 | ABT100 intracellular | Inhibition |
| GABBR1 | GBR1 | Baclofen extracellular region | Activation |
|  |  | SGS742 extracellular region | Inhibition |
| ESR2 | ESR2 | Clomifene intracellular | Unspecified |
|  |  | Estradiol cytoplasm | Activation |
|  |  | Tamoxifen intracellular | Unspecified |
|  |  | Afimoxifene intracellular | Inhibition |
|  |  | Progesterone intracellular | Inhibition |
|  |  | Ethinylestradiol intracellular | Activation |
|  |  | Fulvestrant intracellular | Inhibition |
|  |  | Arzoxifene intracellular | Inhibition |
|  |  | TAS108 intracellular | Activation |
|  |  | Raloxifene intracellular | Inhibition |
|  |  | Prinaberel intracellular | Unspecified |
|  |  | CHF4227 intracellular | Unspecified |
|  |  | Pipendoxifene intracellular | Unspecified |
|  |  | LY2066948 intracellular | Inhibition |
